# Clinical Characteristics and Outcome of Patients With Severe COVID-19 Pneumonia at a Public Sector Hospital in Karachi, Pakistan

**DOI:** 10.7759/cureus.13107

**Published:** 2021-02-03

**Authors:** Shehla Baqi, Arshi Naz, Muneeba Ahsan Sayeed, Samita Khan, Humera Ismail, Vijai Kumar, Hiranand Somjimal, Jahangir Aneela, Sidra Imtiaz, Sadqa Aftab

**Affiliations:** 1 Infectious Diseases, Shaheed Mohtarma Benazir Bhutto Institute of Trauma, Karachi, PAK; 2 Anesthesiology, Shaheed Mohtarma Benazir Bhutto Institute of Trauma, Karachi, PAK; 3 Research Development, Shaheed Mohtarma Benazir Bhutto Institute of Trauma, Karachi, PAK; 4 Pharmacy, Shaheed Mohtarma Benazir Bhutto Institute of Trauma, Karachi, PAK

**Keywords:** pneumonia, intensive care unit, mortality, outcome, complications, pakistan, covid-19

## Abstract

Introduction: In Pakistan, the first case of COVID-19 was reported in February of 2020, cases peaked in June, and by January 2021, approximately 500,000 confirmed cases and over 10,000 deaths have been reported. There is a lack of data in Pakistan of the demographics, clinical characteristics, and outcome of patients with COVID-19 pneumonia, particularly those with severe illness, which we aim to assess.

Methods: This is a single-centered, observational study conducted at the COVID unit of the Shaheed Mohtarma Benazir Bhutto Institute of Trauma in Karachi, Pakistan. A manual medical record review of patients admitted from April 24, 2020 to August 24, 2020 was conducted, and all patients with polymerase chain reaction (PCR) positive for severe acute respiratory syndrome coronavirus 2 (SARS-CoV2) with moderate, severe, and critical COVID-19 pneumonia were included.

Results: Of 299 patients, the median age was 60 years (50-65). Males accounted for 221 (73.9%). Most common symptoms were shortness of breath seen in 270 (90.3%) and fever in 225 (75.3%) patients. Diabetes mellitus (51.2%) and hypertension (50.3%) were the predominant co-morbidities. COVID disease was categorized on admission as moderate in 68 (22.7%), severe in 151 (50.5%), and critical in 80 (26.8%) patients. Survival analysis was done in 252 patients, all of whom received steroids, while tocilizumab was administered to 111 (44%) patients. Hundred (39.7%) patients received non-invasive ventilation (NIV), while 57 (22.6%) were placed on mechanical ventilation. Overall, 95 (37.7%) patients died. Factors associated with mortality included older age with those above 60 years more likely to die (odds ratio [OR]: 1.925; 95% CI: 1.148-3.228; pvalue: 0.009), presence of co-morbidities (OR 1.843; 95% CI: 0.983-3.456; p value: 0.070), development of cytokine release syndrome (CRS) (73 [56.2%] vs 57 [43.8%], p value: <0.001), acute kidney injury (31 [81.6%] vs 7 [18.4%], p value: <0.001), cardiac complications (12 [75%] vs 4 [25%], p value: 0.002), and sepsis (29 [87.9%] vs 4 [12.1%], p value: <0.001). Non-survivors were more likely to develop acute respiratory distress syndrome (ARDS), having been placed on NIV and mechanical ventilation. Laboratory parameters at final outcome found that in non-survivors, median total leukocyte count, C-reactive protein (CRP), neutrophil lymphocyte ratio (NLR), and lactate dehydrogenase (LDH) were higher, while absolute lymphocyte count and platelet counts were lower which were found to be statistically significant compared to survivors.

Conclusion: In this study of patients with severe COVID-19 pneumonia at a public sector hospital in Karachi, Pakistan, most were males, and the average age was 60 years. Mortality was high, and associated factors included older age, presence of comorbid conditions, and the development of ARDS, CRS, and sepsis.

## Introduction

Severe acute respiratory syndrome coronavirus 2 (SARS-CoV-2) infection was first reported in December 2019 in Wuhan, China. By 2021, over 88 million people had developed coronavirus disease (COVID-19), and more than 1.9 million people had died in a pandemic affecting 218 countries and territories [1]. Severe to critical illness from COVID-19 has been reported from China, Italy, USA, and other countries with mortality rates ranging from 16% to 62% [2-6].

In Pakistan, the first case was reported in February of 2020. Cases peaked in June followed by a steep decline by August throughout the country. By mid-November, over 350,000 confirmed cases and 7000 deaths had been reported [1]. Pakistan, despite being a low-income country with a population of over 220 million, poor healthcare infrastructure, and illiteracy, had fared well with far fewer cases and mortality than originally predicted [7]. However, signs of resurgence had become evident by November 2020, and by January 2021, approximately 500,000 confirmed cases and over 10,000 deaths have been reported [1].

The Health Ministry of Sindh designated the Shaheed Mohtarma Benazir Bhutto Institute of Trauma (SMBBIT) as a facility for COVID patients requiring higher level of care. SMBBIT is centrally located in Karachi, the port city of Pakistan, which has an estimated population of over 25 million. Therefore, on April 24, 2020, the COVID unit was established with a 26-bedded ICU and a 28-bedded intermediate care unit (IMCU). The government provided funds for renovations, negative ventilation in ICU, equipment, and medications. Doctors, nurses, and paramedical staff were mobilized from throughout the province of Sindh and also from within the institute and routed to the COVID unit at the Trauma Institute.

There is a lack of data from Pakistan on the demographics, management, and outcome of patients with COVID pneumonia, particularly those with severe to critical illness. The purpose of this study is to generate local data to facilitate a better understanding of our patient population in order to enable early identification of individuals who are at risk of developing severe to critical illness. We aim to assess factors associated with mortality as well as to evaluate the management approaches and supportive therapies that were used so as to improve future outcomes.

## Materials and methods

This is a single-centered, retrospective observational study conducted at the COVID unit of the SMBBIT in Karachi, Pakistan. A manual medical record review of all patients admitted to the COVID unit from April 24, 2020 to August 24, 2020 was carried out. The majority of patients were referred to the SMBBIT COVID unit because of acute hypoxemia requiring respiratory support. Referrals were communicated through a WhatsApp-based group that incorporated six major hospitals of Karachi [8]. Once accepted by the designated COVID Focal Physician at SMBBIT, patients were admitted directly into the unit. Patients with moderate disease were admitted to the intermediate care unit (IMCU) for supplemental oxygenation. Those with severe to critical disease were admitted to the intensive care unit (ICU), defined as a unit with negative pressure ventilation with the capability of providing non-invasive ventilation (NIV), high-flow nasal cannula oxygenation (HFNC), and invasive mechanical ventilation (IMV).

All admitted patients with laboratory confirmed PCR positive for SARS-CoV-2 with moderate, severe to critical COVID pneumonia were included. We excluded those patients who tested negative for SARS-CoV-2 despite suspicion for COVID, had mild COVID not requiring supplemental oxygen having clear chest x-ray, patients whose files were incomplete, and intra-hospital transfers to the COVID unit from within the Trauma Institute whose primary diagnosis was not COVID but tested positive during screening.

Disease classification was according to the National Guidelines [9]. Moderate disease was described as oxygen saturation (SpO2) ≤ 94% but >90% and mild pneumonia. Severe disease was described as one or more symptoms that included a respiratory rate of ≥30/min, SpO2 ≤ 90% on room air, arterial oxygen partial pressure/fractional inspired oxygen (PaO2/FiO2) ratio < 300, lung infiltrates >50% of the lung field within 24-48 hours of admission, altered level of consciousness, convulsions, dehydration, myocardial injury, elevated liver enzymes, or coagulopathy. Critical disease was the presence of acute respiratory distress syndrome (ARDS), multi-organ dysfunction, or septic shock.

We used the World Health Organization (WHO) Six-Category Scale of Clinical Status to map oxygenation support and disposition of patients [10]:1 = Not hospitalized; 2 = Hospitalized, not requiring supplemental oxygen; 3 = Hospitalized, requiring supplemental oxygen; 4 = Hospitalized, requiring HFNC, NIV or both; 5 = Hospitalized, requiring IMV; and 6 = Death.

Hospitalized patients with COVID-19 received supportive care according to the institutional guidelines that had been adapted from the National COVID guidelines [9]. All patients obtained a chest x-ray, whereas CT imaging was not routinely performed. Standard care on admission, unless contraindicated, included intravenous methylprednisone averaging 1mg/kg/day, enoxaparin subcutaneously, IV omeprazole, and azithromycin. Mode of oxygenation, with a target SpO2 of at least 92%, and ventilation strategies in case of ARDS, need for vasopressors, and renal replacement therapy were determined by the primary team delegated by the department of anesthesia. Daily comprehensive Infectious Diseases rounds evaluated patients for onset of cytokine release syndrome (CRS), hospital-acquired bacterial infections, sepsis, and coagulopathy. CRS is defined as any of the following in the presence of moderate, severe, or critical disease [9].

1. Ferritin > 1000 mcg/L and rising in last 24 hours

2. Ferritin > 2000 mcg/L in patient requiring high-flow oxygen or ventilation

3. Absolute lymphocyte count < 800 cells/ml, or neutrophil to lymphocyte ratio of >5 and two of the following:

 a. Ferritin > 700 mcg/mL and rising in the last 24 hours

 b. Lactate dehydrogenase (LDH) > 300 IU and rising in the last 24 hours

 c. D-dimer > 1000ng/mL (or >1 mcg/ml) and rising in the last 24 hours

 d. C-reactive protein (CRP) > 70 mg/L (or >10 high-sensitivity CRP [hs-CRP]) and rising in the last 24 hours, in the absence of bacterial infection

If any three are present on admission, no need to rise document.

Remdesevir was not available in Pakistan at that time. Intravenous immunoglobulin was too costly for the government budget. Tocilizumab was available for CRS on request by Infectious Diseases. All treatment was provided free of cost to all patients.

Data was ascertained by a manual review of the medical records and patient flow sheets. Laboratory and radiological data were abstracted from the electronic data system. Demographics, symptoms at presentation, disease classification, co-morbidities, complications during hospitalization, and outcomes were documented. Baseline data were defined as information obtained within 24 hours of admission to the COVID unit. The first day of admission was deemed Day 0 of the study, and data were abstracted on days 0, 3, 7, 14, 21, 28, 35, 42, and day of discharge, transfer, or death. The study patients were followed for their entire hospitalization.

Primary outcome was mortality. Secondary outcomes included incidence of ARDS, proportion of patients requiring mechanical ventilation, and duration of hospitalization. Characteristics of non-survivors were compared to survivors. Comparisons included demographics, day of illness, and severity of disease at presentation, underlying co-morbidities, laboratory and radiological parameters, mode of oxygenation, and complications. Clinical response to management strategies over the course of hospitalization was gauged by the evolution of laboratory parameters such as CRP and LDH and by the six-category clinical WHO ordinal scale.

This study was a retrospective chart review, so requirement for patient consent was waived by the Institute’s Ethics Review Committee (ERC number-000014/SMBBIT/Approval/2020). Patient confidentiality was maintained, and patient data were kept securely without identifiers such as name and residential address.

Data were entered on the IBM SPSS version 24 (IBM Inc., Armonk, USA). Continuous variables were expressed as medians, interquartile ranges as appropriate. Student t-test was used for categorical data, which was presented as counts and percentages. No imputation was performed for missing data. Comparisons used the chi-squared test or Fischer’s exact test as appropriate. The difference between the survivors and non-survivors was assessed by using the two-sample t-test or Wilcox rank-sum test. Significance was set at α less than 0.05. All statistics were deemed as descriptive only since patients had not been randomly selected.

## Results

**Figure 1:**
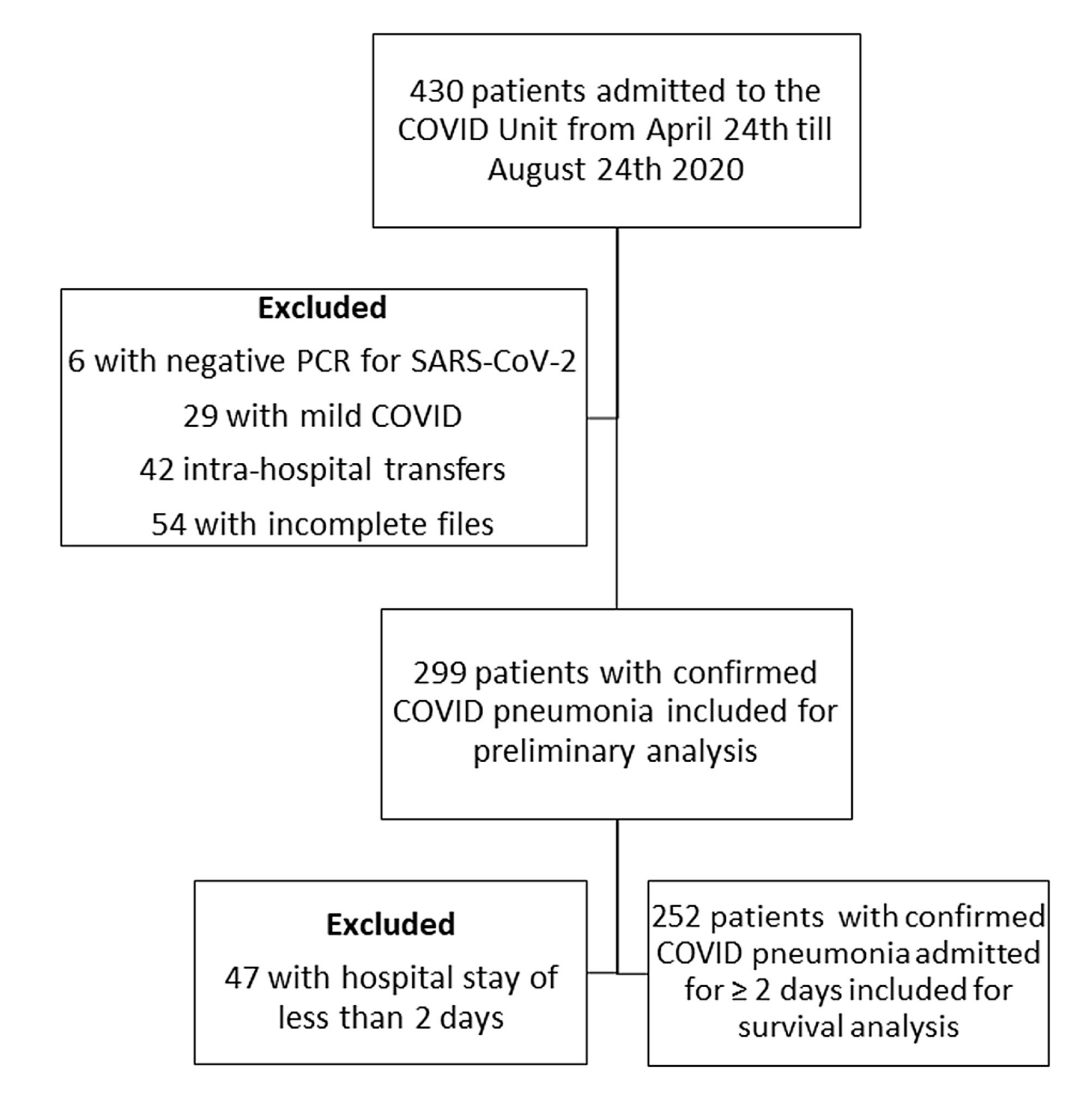
Flow Chart Regarding Selection of Inpatient COVID-19 Cases PCR, polymerase chain reaction; SARS-CoV2, severe acute respiratory syndrome coronavirus 2; COVID, coronavirus disease.

Over the four-month study period, 430 adult patients were admitted to the COVID unit at SMBBIT, of which a total of 131 (30.5%) were excluded (Figure 1).

Demographics and clinical characteristics of 299 patients at baseline, within 24 hours of admission, are shown in Table 1.

**Table 1:** Baseline Characteristics at Presentation and Final Outcome in 299 Patients With COVID-19 Pneumonia IQR, Interquartile range; HFNC, high-flow nasal canula; NIV, non-invasive ventilation.

Demographics	n (%)
Age in years (Median IQR)	60 (50-65)
Male	221 (73.9)
Female	78 (26.1)
Healthcare worker	5 (1.7)
Admitted from	
Home	90 (30.1)
Referral from healthcare facility	209 (69.8)
Symptoms	
Fever	225 (75.3)
Cough	170 (56.9)
Shortness of breath	270 (90.3)
Headache	3 (1.0)
Diarrhea	18 (6.0)
Altered level of consciousness	13 (4.3)
Day of illness since onset of symptoms (Median IQR)	7 (5-10)
COVID-19 Disease Category	
Moderate	68 (22.7)
Severe	151 (50.5)
Critical	80 (26.8)
Inpatient Disposition on Admission	
Intermediate care unit (IMCU)	160 (53.3)
Intensive care unit (ICU)	139 (46.5)
Co-morbidities	222 (74.2)
Diabetes mellitus	153 (51.2)
Hypertension	150 (50.3)
Asthma	11 (3.7)
COPD	4 (1.3)
Congestive heart failure	1 (0.3)
Ischemic heart disease	45 (15.0)
Chronic kidney disease	7 (2.3)
Chronic liver disease	1 (0.3)
Cerebrovascular ischemia	7 (2.3)
Malignancy	1 (0.3)
Obesity	23 (7.7)
Chest X-ray Findings	
Unavailable	11 (3.68)
Clear	2 (0.7)
Unilateral	19 (6.35)
Bilateral	267 (89.3)
Laboratory Findings on Admission (Median IQR)	
Total leukocyte count (TLC) (10^9^/L)	11 (8-14)
Absolute lymphocyte count (ALC) (10^9^/L)	1.1 (0.7-1.5)
Neutrophil lymphocyte ratio (NLR)	8.1 (5.3-13.2)
Hemoglobin (g/L)	13 (11.2-13.7)
Platelets (10^9^/L)	255 (185-334)
Creatinine (mg/L)	0.9 (0.8-1.3)
Sodium (mmol/L)	138 (134-141)
C-reactive protein (CRP) (mg/L) n = 261	118.7 (57.8-219.2)
Lactate dehydrogenase (LDH) (U/L) n = 230	581 (443-737)
Ferritin (mg/L) n = 80	887 (461.7-1361.8)
WHO Six-Category Scale Within 24 Hours of Admission	
1 (not hospitalized)	0 (0)
2 (not requiring supplemental oxygen)	13 (4.3)
3 (requiring supplemental oxygen)	206 (68.9)
4 (requiring HFNC, NIV, or both)*	53 (17.7)
5 (requiring invasive mechanical ventilation)	11 (3.7)
6 (death)	16 (5.4)
Outcome	
Duration of hospitalization days (Median IQR)	7 (4-11)
Final Disposition	
Home	140 (46.8)
Transferred to other healthcare facility	4 (1.3)
Left against medical advice/discharged on request	22 (7.3)
Death	133 (44.5)

The median age was 60 (IQR 50-65), with a range of 19 to 92 years. A total of 221 (73.9%) were males. There were 209 (69.8%) patients who were referred from other healthcare facilities of whom five patients were received intubated and six were intubated within 24 hours of admission. Most frequently reported symptom was shortness of breath in 270 patients (90.3%). Patients presented at a median of seven (IQR 5-10) days since onset of illness. Glasgow Coma Scale was 15/15 in 263 (88%) on admission.

COVID-19 disease was categorized as moderate in 68 (22.7%), severe in 151 (50.5%), and critical in 80 (26.8%) patients. Of 299, 139 (46.5%) were directly admitted to the ICU. Co-morbidities were seen in 222 (74.2%) patients with diabetes mellitus most commonly reported in 153 (51.2%). Patients’ weight and height were not documented, and body mass index (BMI) could not be calculated, but in 23 (7.7%), obesity was noted in the medical records.

Among the 288 patients in whom chest x-ray was available, the most common radiological finding was bilateral infiltrates in 267 (93%). At presentation, median absolute lymphocyte count (ALC) was 1.1 × 109/L (0.13-3.9) with a median neutrophil lymphocyte ratio (NLR) of 8.4 (1.2-47.5). Median CRP was elevated at 119 mg/L (0.8-527), and median LDH was elevated at 581U/L (213-4090).

**Figure 2:**
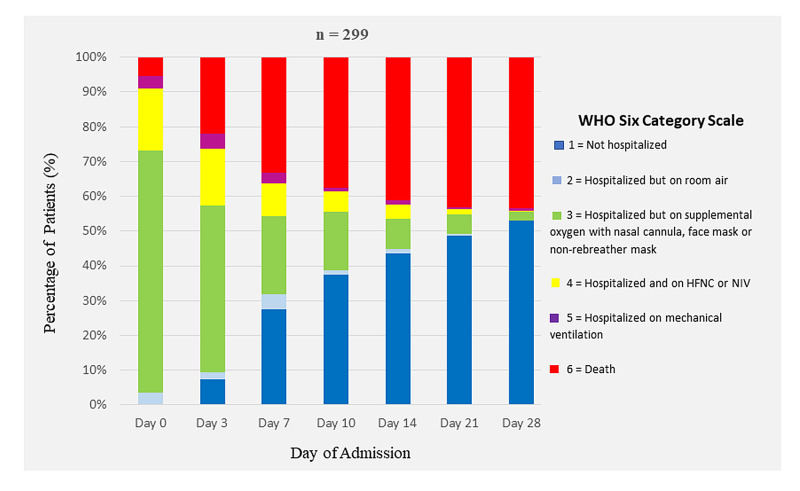
Oxygen Support and Outcome in 299 Patients With COVID-19 Pneumonia WHO, World Health Organization; HFNC, high-flow nasal canula; NIV, non-invasive ventilation.

Median duration of hospitalization was seven (4-11) days. Oxygen support during hospitalization and final disposition of patients, whether discharge or death, in accordance with the WHO six-category scale is shown in Figure 2. Of 299, 133 (44.5%) patients died.

In order to remove bias in comparison between non-survivors and survivors, we excluded 47 (15.7%) patients who were admitted for less than two days, 38 of whom had died, some within hours of admission. Therefore, 252 patients who were admitted for two or more days underwent further analysis, shown in Table 2. Comparison of demographics and clinical characteristics of survivors with non-survivors in 252 patients with COVID-19 pneumonia is shown in Table 2.

**Table 2:** Comparison of Demographics, Clinical Characteristics, and Laboratory Parameters Between Non-survivors and Survivors in 252 Patients With COVID Pneumonia IQR, Interquartile range.

Variables	Total (n = 252) n (%)	Non-survivors (n = 95) n (%)	Survivors (n = 157) n (%)	p value
Demographics				
Median age (IQR) years	60 (50-65)	62 (55-70)	56 (47-64)	0.384
≤60 years of age	142 (56.3)	44 (31.0)	98 (69.0)	0.013
>60 years of age	110 (43.6)	51 (46.4)	59 (53.6)	
Males	188 (74.6)	68 (36.2)	120 (63.8)	0.238
Co-morbidities	190 (75.4)	78 (41.1)	112 (58.9)	0.070
Diabetes mellitus	132 (52.4)	50 (37.9)	82 (62.1)	0.527
Hypertension	128 (50.8)	53 (41.4)	75 (58.6)	0.135
Ischemic heart disease	37 (14.7)	16 (43.2)	21 (56.8)	0.282
Obesity	22 (8.7)	7 (31.8)	15 (68.2)	0.363
Day of illness since onset of symptoms (Median, IQR)	7 (5-10)	7 (4-9.7)	7 (5-10)	0.829
COVID-19 Disease Category at Presentation				
Moderate	60 (23.8)	1 (1.7)	59 (98.3)	<0.001
Severe	133 (52.8)	41 (30.8)	92 (69.2)	
Critical	59 (23.4)	53 (89.8)	6 (10.2)	
Treatment in addition to Standard Care				
Tocilizumab	111 (44.0)	58 (52.3)	53 (47.7)	<0.001
Empiric antibiotics	125 (49.6)	62 (49.6)	63 (50.4)	<0.001
Cytokine Release Syndrome	130 (51.6)	73 (56.2)	57 (43.8)	<0.001
Complications	184 (73.0)	95 (51.6)	89 (48.8)	<0.001
Acute respiratory distress syndrome	97 (38.5)	91 (93.8)	6 (6.2)	<0.001
Acute kidney injury	38 (15.1)	31 (81.6)	7 (18.4)	<0.001
Hospital-acquired pneumonia	66 (26.2)	23 (34.8)	43 (65.2)	0.343
Sepsis	33 (13.1)	29 (87.9)	4 (12.1)	<0.001
Urinary tract infection	19 (7.5)	10 (52.6)	9 (47.4)	0.126
Ischemic heart disease	16 (6.3)	12 (75.0)	4 (25.0)	0.002
Subcutaneous emphysema	6 (2.4)	5 (83.3)	1 (16.7)	0.030
Gastrointestinal bleed	2 (0.8)	2 (100.0)	0 (0.0)	0.141
Vital Signs Within 24 hours of Final Outcome (Median, IQR)				
Highest systolic blood pressure (mm/Hg)	128 (119-140)	130 (107-156)	128 (120-135)	0.448
Oxygen saturation % highest	97 (94-98)	94 (90-98)	98 (96-99)	<0.001
Oxygen saturation % lowest	92 (85-95)	80 (70-88)	94 (92-96)	<0.001
Laboratory Findings Within 24 Hours of Final Outcome (Median, IQR)				
Total leukocyte count (TLC) (10^9^/L)	12.2 (9.1-16.1)	15.4 (10.0-20.7)	11.7 (8.8-13.6)	<0.001
Absolute lymphocyte count (ALC) (10^9^/L)	1.3 (0.9-2.1)	1.1 (0.8-1.7)	1.4 (1.0-2.1)	<0.001
Neutrophil lymphocyte ratio (NLR)	7.3 (4.2-12.8)	12.6 (6.8-22.8)	5.7 (3.7-9.1)	<0.001
Platelets (10^9^/L)	296 (211-387)	225 (160-312)	336 (260-435)	<0.001
Creatinine (mg/L)	0.8 (0.6-1.3)	1.7 (0.8-3.8)	0.7 (0.6-0.9)	<0.001
Sodium (mmol/L)	139 (8.5-144)	146 (139-154)	137 (135-139)	<0.001
C-reactive protein (CRP) (mg/L) (n = 228)	16 (4.8-54.9)	58 (20-133.7)	8.4 (3.5-24.1)	<0.001
Lactate dehydrogenase (LDH) (U/L) (n = 206)	48 (342-819)	846 (632-1098)	361.5 (314-490)	<0.001
Ferritin (mg/L) (n = 12)	912.5 (413-1998.2)	2008 (879-3688)	707 (363-1043)	0.088
Outcome				
Intensive care unit stay (ICU)	166 (65.9)	93 (56.0)	73 (44.0)	<0.001
ICU stay in days (Median, IQR)	6 (4-10)	6 (4-9)	7 (4-11)	0.530
Non-invasive ventilation (NIV)	100 (39.7)	72 (72.0)	28 (28.0)	<0.001
High-flow nasal cannula (HFNC)	21 (8.3)	10 (47.6)	11 (52.4)	0.226
Invasive mechanical ventilation (IMV)	57 (22.6)	53 (93.0)	4 (7.0)	<0.001
Number of days on IMV (Median, IQR)	3 (1-4)	3 (1-4)	2 (1-3)	0.798
Median hospitalization days (Median, IQR)	8 (5-13)	7 (4-11)	8 (6-15)	0.001

Radiological findings

The most common pattern seen on initial chest x-ray was bilateral patchy or interstitial infiltration seen in 230 (91.3%) patients. Of 252, 20 (7.9%) patients had CT scan chest imaging during the course of their hospitalization, of which 11 (55%) demonstrated fibrosis and 16 (80%) had ground glass opacities.

Management

All patients with hypoxemia received steroids and prophylaxis for thromboembolic disease as part of standard of care. The mean duration of steroids during hospitalization was seven (1-37) days. Of 157 patients who left hospital, 38 (24.2%) were prescribed a tapering dose of oral steroids and 54 (34.4%) received rivaroxaban. All patients received azithromycin and omeprazole.

Antibiotics were prescribed, either empirically or targeted, for hospital-acquired infections, which consisted of meropenem in 110 (43.6%) patients, which was given in combination with colistin in 40 (15.8%) patients, vancomycin in 23 (9.1%) patients, and voriconazole in two (0.79%) patients. Of 252 patients, CRS was seen in 130 (51.6%) of whom tocilizumab was administered to 111 (44%) patients of whom 52.3% died.

Complications

Of 252 patients, 184 (73%) had complications of COVID, the most common of which was ARDS in 97 (38.5%). Acute kidney injury occurred in 38 (15.1%) during hospitalization. Sixty-six (26.2%) patients developed bacterial pneumonia, and 33 (13.1%) developed sepsis. Subcutaneous emphysema was seen in six patients (2.4%), five of whom were on NIV, and two had pneumothorax.

Outcome

Of 252, 166 (65.9%) patients had ICU disposition during their hospitalization with a median duration of ICU stay of six (4-10) days. Of these, 100 (39.7%) patients received NIV for a median of four (2-6) days, and of these, 38 (38%) subsequently went onto mechanical ventilation of whom 37 died. Of 252, 21 (8.3%) patients were on HFNC for a median of three (1-4) days of whom five patients (23.8%) went onto mechanical ventilation and all five of them died.

Overall, of 252 patients, 57 (22.6%) were placed on mechanical ventilation for a median of three (1-4) days, and 53 (93%) died. The mean age of those on mechanical ventilation (MV) was 60 (34-80) years. Median duration of hospitalization was eight (5-13) days. Patients who developed fibrosis as reported on CT scan had a prolonged duration of mean stay of 20 (4-40) days. Overall, of 252 patients, 95 (37.7%) died. Of 166 patients who had ICU stay, 93 (56%) died. Of 60 patients categorized with moderate disease at presentation, one died.

Comparison between non-survivors and survivors is shown in Table 2. Factors associated with mortality included older age with those above 60 more likely to die (odds ratio [OR]: 1.925; 95% CI: 1.148-3.228; p value = 0.009) and presence of one or more co-morbidities (odds ratio [OR]: 1.843; 95% CI: 0.983-3.456; p value = 0.070). Mortality was associated with the development of CRS (73 [56.2%] vs 57 [43.8%] p value < 0.001), acute kidney injury (31 [81.6%] vs 7 [18.4%] p value < 0.001), cardiac complications (12 [75%] vs 4 [25%] p value 0.002) and sepsis (29 [87.9%] vs 4 [12.1%] p value < 0.001).

Non-survivors, when compared with survivors, were more likely to have developed complications of COVID-19, which included ARDS (91 [93.8%] vs 6 [6.2%] p value < 0.001) and were more likely to have been placed on NIV (72 [72%] vs 28 [28%] p value < 0.001) and mechanical ventilation (53 [93%] vs 4 [7%] p value < 0.001). On day of final outcome, those who died compared to those who were discharged had significantly lower median oxygen saturations with the highest achieved at 94% compared with 98% with p value < 0.001 and the lowest at 80% compared with 94% with p value < 0.001.

**Figure 3:**
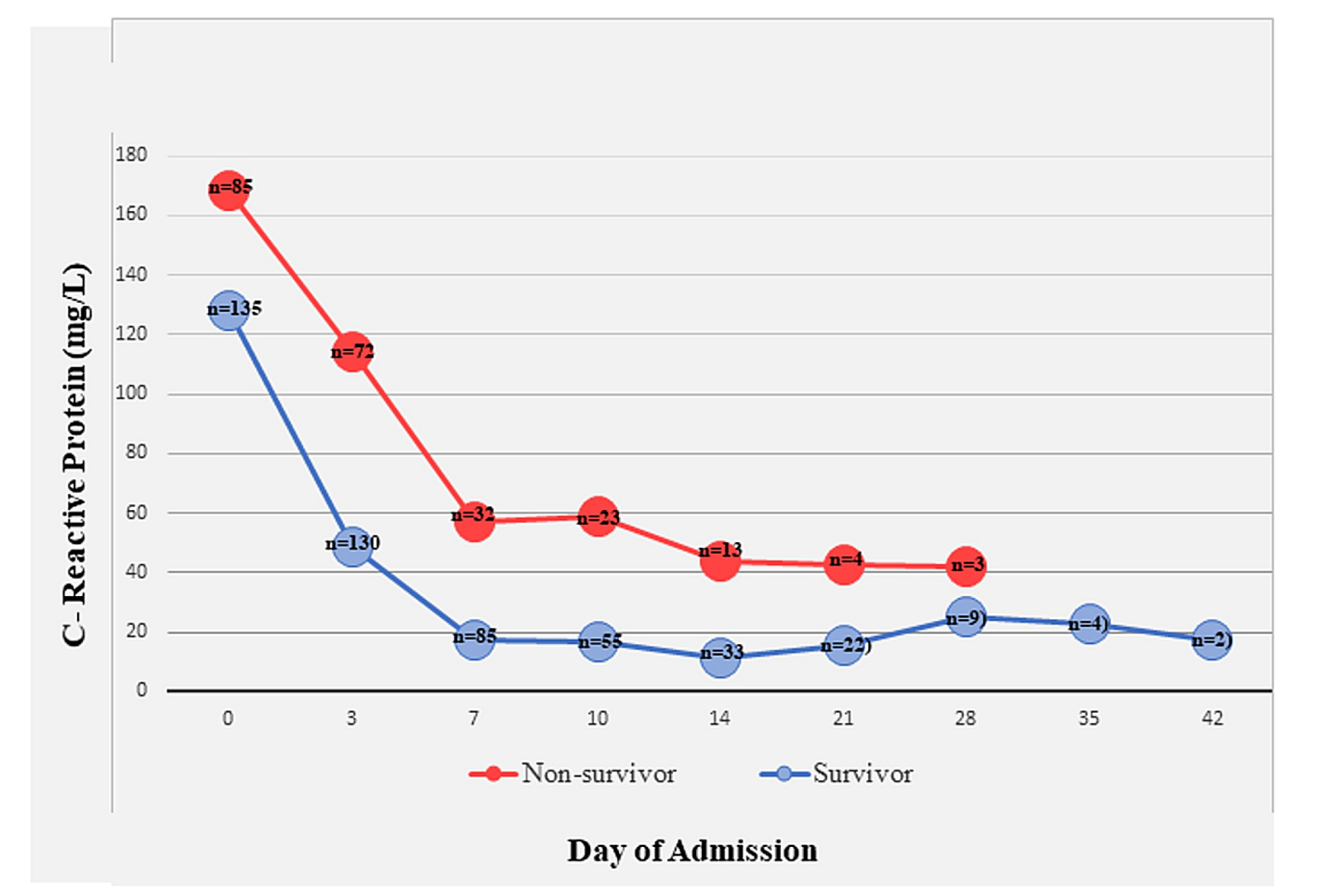
Temporal Changes in C-Reactive Protein in Non-survivors and Survivors Among 252 Patients With COVID Pneumonia

Laboratory parameters documented within one day of death or discharge were compared between non-survivors and survivors. In non-survivors, median total leukocyte counts were higher (15.4 vs 11.7 × 109/L p value < 0.001), absolute lymphocyte counts were lower (1.1 vs 1.4× 109/L p value < 0.001), NLRs were higher (12.6 vs 5.7 p value < 0.001), and platelets were lower (225 vs 336 × 109/L p value < 0.001) than those in survivors. Serum sodium was higher in those who died (146 vs 137 mmol/L p value < 0.001). LDH was higher in non-survivors (846 vs 361 U/L p value < 0.001). Median CRP was higher in non-survivors (58 vs 8.4 mg/L p value < 0.001). Temporal changes in CRP over the course of hospitalization in non-survivors and survivors is plotted in Figure 3.

## Discussion

This study has provided a better understanding of our COVID-19 patient population baseline characteristics, hospital course, and clinical outcomes. We found that patients admitted to the COVID intermediate and intensive care units were predominantly males as reported in numerous studies, including from Pakistan [2-5,11]. Studies of COVID-19 patients have shown that males have a higher risk of developing severe form of disease with higher fatality rate than female patients [12].

We reported a median age of 60 years, which is much lower than that reported from Europe and the United States for patients with severe COVID-19 disease. Pakistan has a population of approximately 220 million, and the median age is 22.8 years [1]. Less than 5% of the population is ≥ 65 years of age, and life expectancy averages 67.8 years [1]. Our data is a reflection of a developing country with a young population that has a diminished life expectancy as compared with the Global North.

A high rate of co-morbidities has been reported in several studies of ICU patients and has been associated with poorer outcomes as also demonstrated in our study [3-5,13]. Individuals with obesity are at increased risk of COVID-19 with increased morbidity and mortality [13,14]. Though obesity was documented in a significant number of our patients, we have likely underestimated it, given that patient weights were not measured.

It is now proposed to classify COVID-19 into simply severe and non-severe by which criteria most of our study patients would be categorized as having severe COVID. Only one of our patients that we categorized as having moderate COVID disease died, which suggests that disease classification into mild, moderate, severe, and critical COVID may be of greater prognostic value.

Steroids were given to all our patients and the recovery Trial has indeed demonstrated that the use of dexamethasone lowers 28-day mortality [15]. Tocilizumab was not associated with a reduction in mortality in our study. Randomized clinical trials of hospitalized adult patients with COVID-19 pneumonia have dampened enthusiasm for tocilizumab, and in one study of patients with Pao2/Fio2 ratio between 200 and 300 mm Hg, no benefit of tocilizumab on disease progression was observed compared with standard care [16]. Further studies are required to define subset of patients and stage of disease where response to tocilizumab may be anticipated. Azithromycin was included in our standard of care protocol, but studies have shown that adding azithromycin does not improve clinical outcomes in COVID pneumonia patients [17]. Therefore, we now recommend that antibiotics be reserved for secondary bacterial infections.

We found an incidence of 15.1% of acute kidney injury (AKI), whereas in critically ill patients, AKI has been reported in 29% by Yang et al. from China. In two studies from New York city of COVID ICU patients, AKI was found in 40.7% on admission and subsequently developed in 36.0% of patients, and in 78% in another study [4,18].

We had a higher incidence of hospital-acquired secondary infections as compared with other studies that reported 4.7% and 6.8% from Barcelona and Wuhan, respectively, associated with poorer outcomes [19,20]. Despite higher numbers, we believe that we may still have underestimated secondary infections since procalcitonin and fungal antigen tests were not readily available. A high patient burden with chronic understaffing during the peak months of COVID, together with the constraints of personal protective equipment, may have contributed to breach of good infection control practices with an increase in hospital-acquired infections.

Hypernatremia in patients who died was possibly contributed by insensible losses in a warm and humid ICU environment, since installation of displacement/negative pressure in the ICU had led to thermal compromise during the peak summer months when this study spanned.

Subcutaneous emphysema was a significant complication, also described in other studies, and has been associated with a poor prognosis [21]. Most of our patients with subcutaneous emphysema were on NIV, and interestingly, only two had concomitant pneumothorax.

Our study found that NIV failed in preventing mechanical ventilation in 38% as compared with 28% in a study by Manna et al. who reported that patients who failed NIV were older, had a higher respiratory rate, PaCO2 and D-dimer levels before NIV, and higher minute ventilation and ventilatory ratio on the first day of NIV [22]. In our study, patients receiving HFNC were few in number, and since we employed an HFNC/NIV alternating strategy on most patients, comparison between the two modalities was not feasible. A review recommends that HFNC could be offered for SARS-CoV-2 patients with PaO2/FiO2 between 200 and 300 and NIV for those with PaO2/FiO2 between 100 and 200 and suggests coupled (HFNC/NIV) strategy [23]. Self-proning was encouraged and facilitated in our patients who were not intubated, but it was not manageable in intubated ICU patients due to shortage of staff. Once intubated, our mortality was very high.

Indeed, the in‐ICU mortality from COVID‐19 is higher than usually seen in ICU admissions with other viral pneumonias. We found a high ICU mortality of 56%. When compared with other studies of COVID pneumonia patients with severe disease, 61.5% had died at 28 days in a study in May 2020 from Wuhan, China [4]. As the pandemic has progressed, the reported mortality rates in patients admitted to the ICU have fallen from above 50% to close to 40%, as demonstrated in a meta-analysis of 24 observational studies from centers across Asia, Europe, and North America [24]. A July 2020 study of 1591 patients admitted to the ICU of the Lombardy region in Italy documented a lower ICU mortality of 26% [25]. In India, a mortality of 18.5% in ICU patients was documented [26]. An observational study in the United Kingdom reported that South Asian ethnicity was associated with an increased risk of death, after adjusting for age, sex, co-morbidities, and health-seeking behaviors, reflecting a more aggressive disease course in these patients [27].

As COVID-19 spreads across the world, with an alarming resurgence in many countries, we must be prepared to receive patients requiring higher level of care for management of acute hypoxemic respiratory failure. Policy-makers must focus not only on an increase in critical care bed capacity, purchasing of equipment and ventilators, and costly air-ventilation strategies but also on addressing the acute shortage of nurses, respiratory therapists, intensivists, and pulmonologists [28]. Investment in human resource must be prioritized [29].

## Conclusions

In this study of patients with severe COVID-19 pneumonia at a public sector hospital in Karachi, Pakistan, most were males, and the average age was 60 years. Mortality was high, and associated factors included older age, presence of comorbid conditions, and the development of complications of ARDS, CRS, and sepsis. Compared to survivors, non-survivors had lower ALC; higher TLC, NLR, CRP, LDH; and serum sodium, which was statistically significant. We need to be prepared for the resurgence of SARS-CoV-2 infection in our population.
